# Genome-Wide Identification and Analysis of the MADS-Box Gene Family in American Beautyberry (*Callicarpa americana*)

**DOI:** 10.3390/plants10091805

**Published:** 2021-08-30

**Authors:** Tareq Alhindi, Ayed M. Al-Abdallat

**Affiliations:** 1 Department of Biological Sciences, School of Science, The University of Jordan, Amman 11942, Jordan; 2 Department of Horticulture and Crop Science, School of Agriculture, The University of Jordan, Amman 11942, Jordan; a.alabdallat@ju.edu.jo

**Keywords:** bioinformatics, genome-wide assay, MADS-box genes, medicinal plant, plant transcription factors

## Abstract

The MADS-box gene family encodes a number of transcription factors that play key roles in various plant growth and development processes from response to environmental cues to cell differentiation and organ identity, especially the floral organogenesis, as in the prominent ABCDE model of flower development. Recently, the genome of American beautyberry (*Callicarpa americana*) has been sequenced. It is a shrub native to the southern region of United States with edible purple-colored berries; it is a member of the *Lamiaceae* family, a family of medical and agricultural importance. Seventy-eight MADS-box genes were identified from 17 chromosomes of the *C. americana* assembled genome. Peptide sequences blast and analysis of phylogenetic relationships with MADS-box genes of *Sesame indicum*, *Solanum lycopersicum*, *Arabidopsis thaliana*, and *Amborella trichopoda* were performed. Genes were separated into 32 type I and 46 type II MADS-box genes. *C. americana* MADS-box genes were clustered into four groups: MIKC^C^, MIKC*, Mα-type, and Mγ-type, while the Mβ-type group was absent. Analysis of the gene structure revealed that from 1 to 15 exons exist in *C. americana* MADS-box genes. The number of exons in type II MADS-box genes (5–15) greatly exceeded the number in type I genes (1–9). The motif distribution analysis of the two types of MADS-box genes showed that type II MADS-box genes contained more motifs than type I genes. These results suggested that *C. americana* MADS-box genes type II had more complex structures and might have more diverse functions. The role of MIKC-type MADS-box genes in flower and fruit development was highlighted when the expression profile was analyzed in different organs transcriptomes. This study is the first genome-wide analysis of the *C. americana* MADS-box gene family, and the results will further support any functional and evolutionary studies of *C. americana* MADS-box genes and serve as a reference for related studies of other plants in the medically important *Lamiaceae* family.

## 1. Introduction

Mints (Lamiaceae) are the sixth largest family of flowering plants and include many ornamental, medical, and edible species, such as basil, rosemary, thyme, peppermint, and spearmint. Full genome and transcriptome sequencing data that are available at the Mints Genome Project database (http://mints.plantbiology.msu.edu/index.html; accessed on 1 July 2021) and separate other projects are enhancing our understanding of this important medical plant family. American beautyberry (*Callicarpa americana*) is known for its prominent purple fruit, and it has been reported that native Americans have used it as an insect repellent and medicinal plant [1]. Studies have revealed a number of terpenoids, such as spathulenol, intermedeol, and callicarpenal that have been isolated from the plant, and proved to be effective as a mosquito repellent in laboratory experiments [2,3]. *Callicarpa* is a representative from the early-diverging mint lineage, and thus, it has an important phylogenetic position to study the evolution of key gene families, such as the MADS-box genes. Recently, the full genome sequence of *C. americana* has been published [4], providing the opportunity to conduct a comprehensive analysis of the *C. americana* MADS-box gene family. However, the identity and function of MADS-box genes in this species have not been reported in detail.

The MADS-box transcription factor family is of key importance; it can be found in almost all eukaryotes, from protists to animals, but in plants, it is most important for its major role in organ identity and cell differentiation from roots to flower development and fruit ripening and, thus, has been extensively studied [5,6,7]. Understanding the genes that regulate flower, root, and fruit development is of key importance, on a scientific fundamental level, as well as on an economic level. The MADS-box gene family also has a role in plants’ developmental plasticity and responses to abiotic stress such as drought, salinity, extreme temperatures, and nutrient deficiency [8,9]. The acronym MADS represents the first letters of its founding members: mini chromosome maintenance 1 (MCM1) of yeast (*Saccharomyces cerevisiae*), agamous (AG) of *Arabidopsis thaliana*, deficiens (DEF) of snapdragon (*Antirrhinum majus* L.), and serum response factor (SRF) of humans [10]. All MADS-box proteins are characterized by the presence of about 60 amino acids long, DNA-binding domain, known as the MADS-box domain (M-domain), located at the N-terminal region of the proteins. The development of the floral organ is controlled by major groups of MADS-box genes, through the ABCDE model of flower development. In this model, tetramers from different subgroups determine the organ identity; sepal development is directed by the A subfamily genes, petal development requires A and B genes, and carpel development is determined by C genes, whereas stamen development is determined by B and C genes. While the D-functional genes are needed in ovule development [11,12,13,14], and the E-functional genes—acting as the glue that binds different members in the tetramer quartet—are required for the development of all floral organs [15,16].

According to majority of studies, M-type (type I) and MIKC-type (type II) are the two evolutionary lineages of MADS-box genes [17,18]. Both types contain the DNA-binding M-domain. The MIKC-type contains several other conserved domains in addition to the M-domain: an intervening (I) domain, a keratin-like (K) domain, and a C-terminal (C) domain [19,20]. Each of these domains has a role in protein–protein interaction with other MADS-box protein forming dimers and tetramers and with non-MADS proteins [21]; in addition, the C-domain is the most variable, and usually, it contains a transcriptional activation domain [13].

The MIKC type II genes can be further classified as MIKC^C^ (C for “classic”) and MIKC*. The MIKC^C^ type is more diverse, containing thirteen subgroups based on structural differences: SQUAMOSA [SQUA (A)], DEFICIENS/GLOBOSA [DEF/GLO (B)], AGAMOUS [AG (C/D)], SEPALLATA [SEP (E)], AGAMOUS-like; AGL6, AGL12, AGL15, AGL17 (ANR1), B sister (Bsis), SUPPRESSOR OF OVEREXPRESSION OF CO 1 [TM3/SOC1], STMADS11 (SVP), FLOWERING LOCUS C [FLC], and TOMATO MADS 8 [TM8]. While the MIKC* is less diverse and has only two subgroups MIKC*-S and MIKC*-P. Studies showed that the MIKC* type has more conserved functions compared to the M-type and MIKC-type through plants evolution [15,18,22]. MIKC*-type genes play an essential role in the development of the male gametophyte in *A. thaliana*, and they have high degree of functional redundancy. The M-type group usually does not contain the K-domain and overall lacks the domains complexity found in MIKC-type proteins. The M-type (type I) genes are divided into three subgroups: Mα, Mβ, and Mγ subgroups in most plants [23].

In this study, the MADS-box gene family for *C. americana* (American beautyberry) has been systematically analyzed. A total of 78 MADS-box genes were identified in 17 chromosomes. These genes were renamed *CamMADS1* to *CamMADS78* based on their locations on the chromosomes, and a phylogenetic tree of all *CamMADS* genes have been constructed. In addition to *C. americana*, the type I and type II MADS-box genes of *Arabidopsis thaliana*, *Sesamum indicum*, *Solanum lycopersicum*, and *Amborella trichopoda* were analyzed and utilized to construct two phylogenetic trees, one for type I and one for type II of these genes. The gene structure and conservative domain in these genes were identified, then the expression patterns of *C. americana* MADS-box genes in various tissues were analyzed. In addition, *cis*-regulatory elements were analyzed and identified in the 2 kb upstream promoter regions. Results indicated their broad range of functions in several *C. americana* tissues, with major roles in flower and fruit development and abiotic stress response. This study will help in improving our understanding of the evolution and function of this essential transcription factor family, in the medically important *Lamiaceae* family [24].

## 2. Results

### 2.1. Identification of MADS-Box Genes and Their Distribution in C. americana Genome

Seventy-eight non-redundant MADS-box genes were obtained using the HMMER toolkit [25] to search the hidden Markov model of the MADS-box DNA-binding domain in *C. americana* proteome sequence, using both SRF (type I) and MEF2 (type II) MADS-box domain sequences (Table 1). Putative MADS-box genes were submitted to the SMART [26] and PROSITE [27] websites for further verification of the presence of the MADS domain. The following *C. americana* MADS-box genes (*CamMADS 9*, *11*, *27*, *35*, *38*, 47, *60*, *61*, *68*, *77*) were identified as type II (MIKC) but they lacked the K-domain. Thus, a further inspection of the genomic data was necessary, and functional sites identification and genome annotation have been carried out using the FGENESH suite [28], using the *CamMADS* genomic DNA in reference to the *S. indicum* genes. The correct exons have been predicted, and the new *CamMADS* annotations contained both the M- and K-domains, as expected.

Each MADS-box protein was then verified by BLASTP function at the Plant Transcription Factor Database [29,30] separately against *A. thaliana, S. lycopersicum* and *S. indicum* and finally against all species. CamMADS proteins were then initially categorized into type I (M-type) and type II (MIKC) based on their homology with their identified orthologues; Table 1 includes *A. thanliana* type II MADS-box genes orthologs, while type I CamMADS have low identity alignment scores to *A. thaliana* to be confidently assigned a specific ortholog. As reported in previous studies of other species, the number of type II MADS-box genes was higher than that of type I MADS-box genes. Five MADS-box proteins were neutral, with pI values between 6.5 and 7.5, 18 were acidic, with pI values less than 6.5, 55 were alkaline, with pI values greater than 7.5. It is worth noting that the average pI for M-type proteins was 7.5, and for the MIKC^C^ group, it was 8.5 (basic), while all MIKC* proteins had acidic pI values, with a 5.6 average. The average predicted molecular weight for M-type proteins was 30,261.9 Da, and for MIKC^C^, 27,866.9 Da, while the MIKC* proteins had the highest molecular weight average of 40226.7 Da. The number of exons in *CamMADS* genes ranged from 1 to 15 exons. The number of exons in type II MADS-box genes (7–15) greatly exceeded the number in type I genes (1–7). The number of exons within the same subgroup did not vary much, with few exceptions.

**Figure 1:**
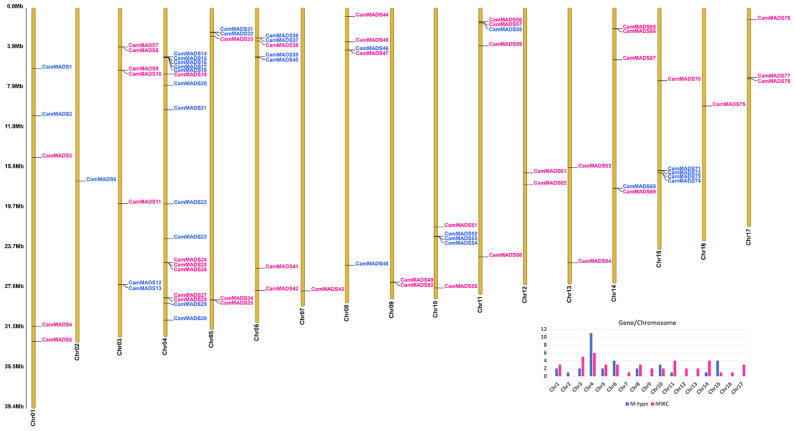
Chromosomal localization of the seventy-eight *C. americana* MADS-box genes. The number of each chromosome is given under the lines, blue indicates M-type genes, and pink indicates MIKC-type. The right side of each chromosome is related to the approximate physical location of each MADS-box gene. Bottom right: a plot of the number and type of *CamMADS* genes per chromosome.

After verification of the presence of the MADS-box domain and initial homology alignments, genes were mapped onto the 17 chromosomes of *C. americana* genome, and no MADS-box genes were located on the unanchored scaffolds (Figure 1). The distribution of *C. americana* MADS-box genes (*CamMADS*) was uneven. The maximum number of genes (17; 21.79%) was localized on Chromosome 4, whereas Chromosomes 2, 7, and 16 have only one MADS-box gene each. Several *CamMADS* genes resided in a 2–5 genes cluster. To investigate possible gene duplication events, the OrthoFinder algorithm was utilized [31], and each two or more adjacent homologous genes located on a single chromosome were considered as co-linear duplicates. A total of 15 paralogous gene pairs were observed, of which four genes were MIKC-type (*CamMADS* 9, 10, 34, 35) and eleven were M-type (CamMADS 20, 21, 39, 40, 52, 53, 54, 71, 72, 73, 74).

### 2.2. Phylogenetic Analysis of MADS-Box Genes in C. americana

**Figure 2:**
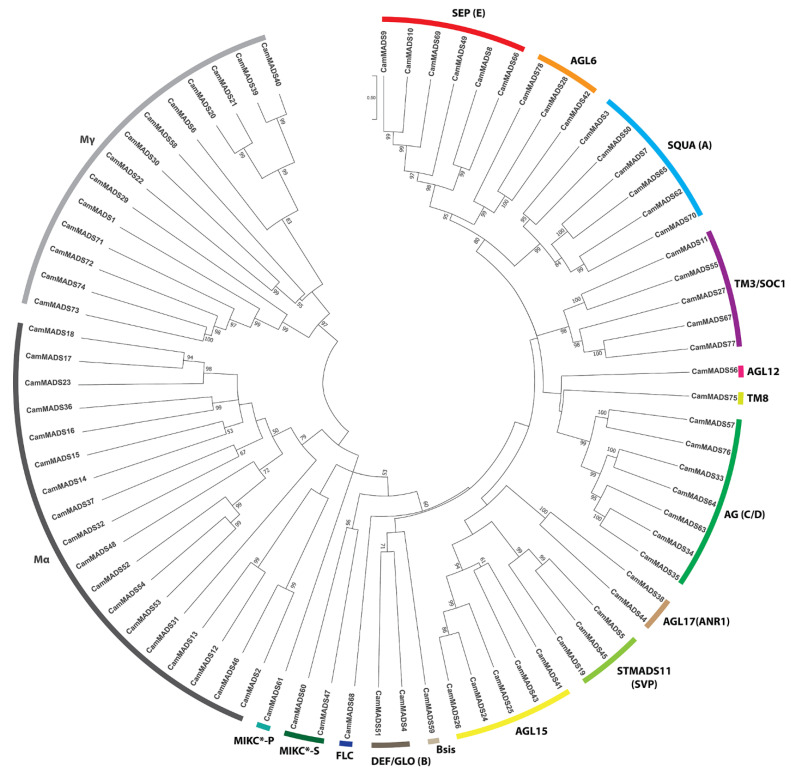
Maximum likelihood tree of CamMADS proteins in *C. americana*. The tree shows type I subgroups (Mα-type, Mγ-type), while the type I Mβ-type group was absent. Type II subgroups; MIKC^C^ (SQUA (A), DEF/GLO (B), AG (C/D), SEP (E), AGL6, AGL12, AGL15, AGL17 (ANR1), Bsis, TM3/SOC1, STMADS11 (SVP), FLC, and TM8. The MIKC* subgroups (MIKC*-S and MIKC*).

**Figure 3:**
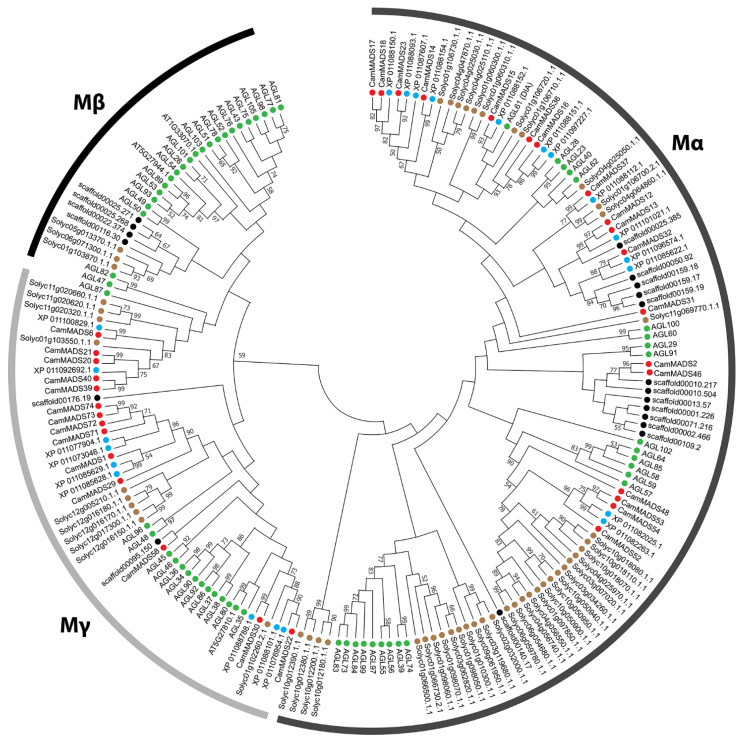
Maximum likelihood tree of type I MADS-box gene proteins in *C. americana, A. thaliana, S. lycopersicum, S. indicum*, and *A. trichopoda*. The MADS-box proteins contained in the branches for each species are indicated by different colored circles: red, *C. americana*; green, *A. thaliana*; brown, *S. lycopersicum*; blue, *S. indicum;* black, *A. trichopoda*. For type I MADS-box proteins ML tree with branch length scale, see Supplementary Figure S1.

**Figure 4:**
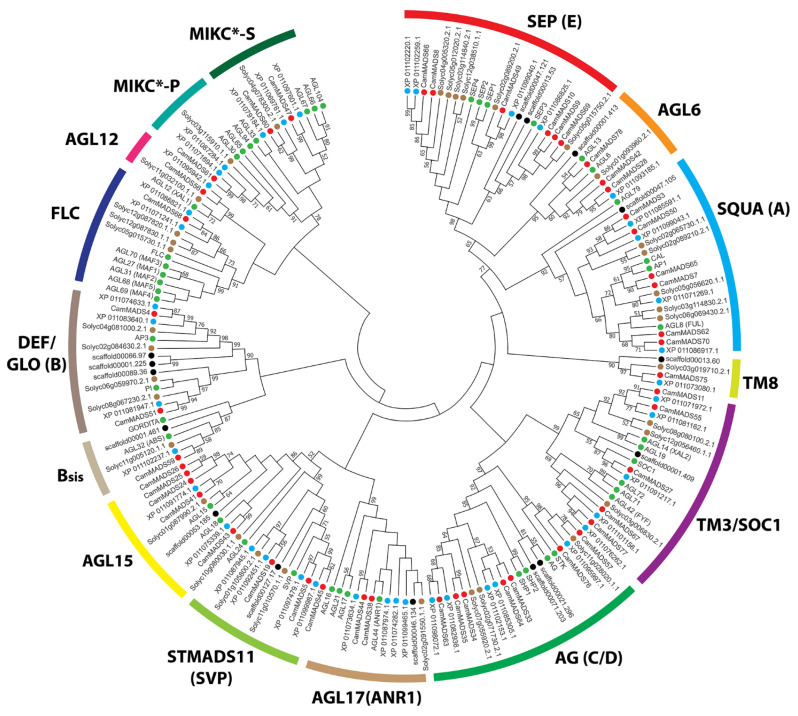
Maximum likelihood tree of type II MADS-box proteins in *C. americana, A. thaliana, S. lycopersicum, S. indicum*, and *A. trichopoda*. The MADS-box proteins contained in the branches for each species are indicated by different colored circles: red, *C. americana*; green, *A. thaliana*; brown, *S. lycopersicum*; blue, *S. indicum;* black, *A. trichopoda*. For type II MADS-box proteins ML tree with branch length scale, see Supplementary Figure S2.

To properly classify the CamMADS proteins, three phylogenetic trees (maximum likelihood (ML) tree) for (1) all identified CamMADS proteins (Figure 2), (2) for type I (Figure 3), and (3) for type II (Figure 4) MADS-box proteins were constructed using type I and type II MADS-box full length proteins from *A. thaliana, S. lycopersicum* (species with well characterized *MADS-box* genes), *S. indicum (a species in lamiaceae family), A. trichopoda* (a basal outer group), and the CamMADS proteins identified in the present study. CamMADS proteins were classified into functional groups according to both *A. thaliana* and *S. lycopersicum* MADS-box genes that have been investigated extensively [32,33]. Based on the phylogenetic tree and structural features of the MADS-box proteins, genes were separated into 46 type II and 32 type I MADS-box genes. *CamMADS* genes were clustered into four groups: type II (MIKC^C^, MIKC*) and type I (Mα-type, Mγ-type), while the type I Mβ-type group was absent (Figure 3). The Mβ-type group was also absent in *S. indicum* (sesame) [34]. While type II (Figure 4) phylogenetic tree included all expected groups and subgroups, in accordance with *A. thaliana* and *S. lycopersicum* trees.

### 2.3. Conservative Motif Distribution and Gene Structure Analysis of C. americana MADS-Box Genes

**Figure 5:**
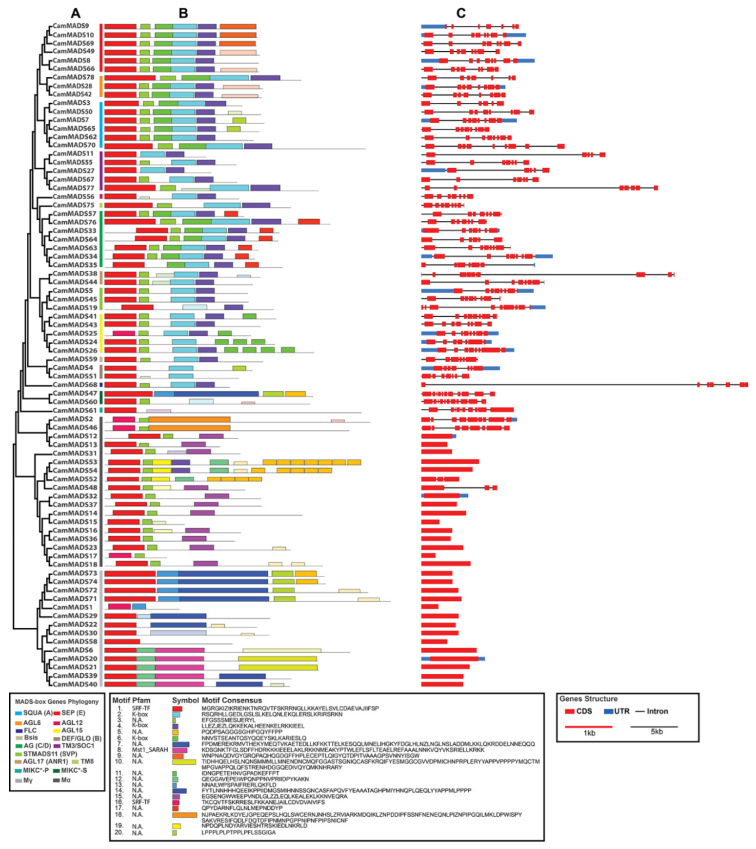
Gene structure and conserved motif analysis of *C. americana* MADS-box proteins: (**A**) CamMADS proteins phylogenetic relationship, (**B**) motif analysis of CamMADS proteins, each motif is represented by a number in a colored box mid-bottom. Box length corresponds to motif length, (**C**) *CamMADS* gene structure analysis (exons are in red, introns represented by solid line, and untranslated regions are in blue).

To better analyze the sequence characteristics and structural differences among the conserved motifs of all CamMADS proteins (list of CamMADS peptide sequences is available in the Supplementary Data), motifs were predicted by the MEME program (Figure 5B). Motifs 1 and 16 represent the DNA binding MADS domain, and Motif 1 was the most typical MADS domain, 50 amino acids in length. Motifs 2 and 4 combined were the highly conserved K-domain (spanning K1, K2, and K3 subdomains). These motifs were present in all MIKC-type *CamMADS* genes. It is worth noting that even when the MEME suite did not recognize some K-domains in few CamMADS proteins, a second check by SMART and MotifFinder suites was enough to confirm that the K-domain was present. The length of the conserved K-domain (K1 + K2 + K3) was 67 (38 + 29) amino acids. In general, CamMADS proteins of the same subgroup had similar motifs, and it is probable that they might have conserved functions. While, the difference in motifs structure and distribution support the expected variety of function of *CamMADS* genes in different organs of *C. americana*.

To gain insights into the structural diversity of *C. americana* MADS-box genes, we analyzed the exon–intron organization of the coding sequences of each *CamMADS* gene (Figure 5C). The number of exons followed a clear bimodal pattern. The type II (MIKC) *CamMADS* all had at least five introns (*CamMADS11* and *27* in TM3/SOC1 group), up to 15 exons (*CamMADS3* in SQUA group). While all type I (M-type) had only one exon—no introns—except, in the Mα group where *CamMADS48* and *52* had three exons, and *CamMADS46* and *2* had 8 and 9 exons, respectively, and in the Mγ group with *CamMADS58* having seven exons. Few genes in the MIKC group have relatively long introns (>10 kb), compared to the rest of *CamMADS* genes.

### 2.4. Expression of C. americana MADS-Box Genes

**Figure 6:**
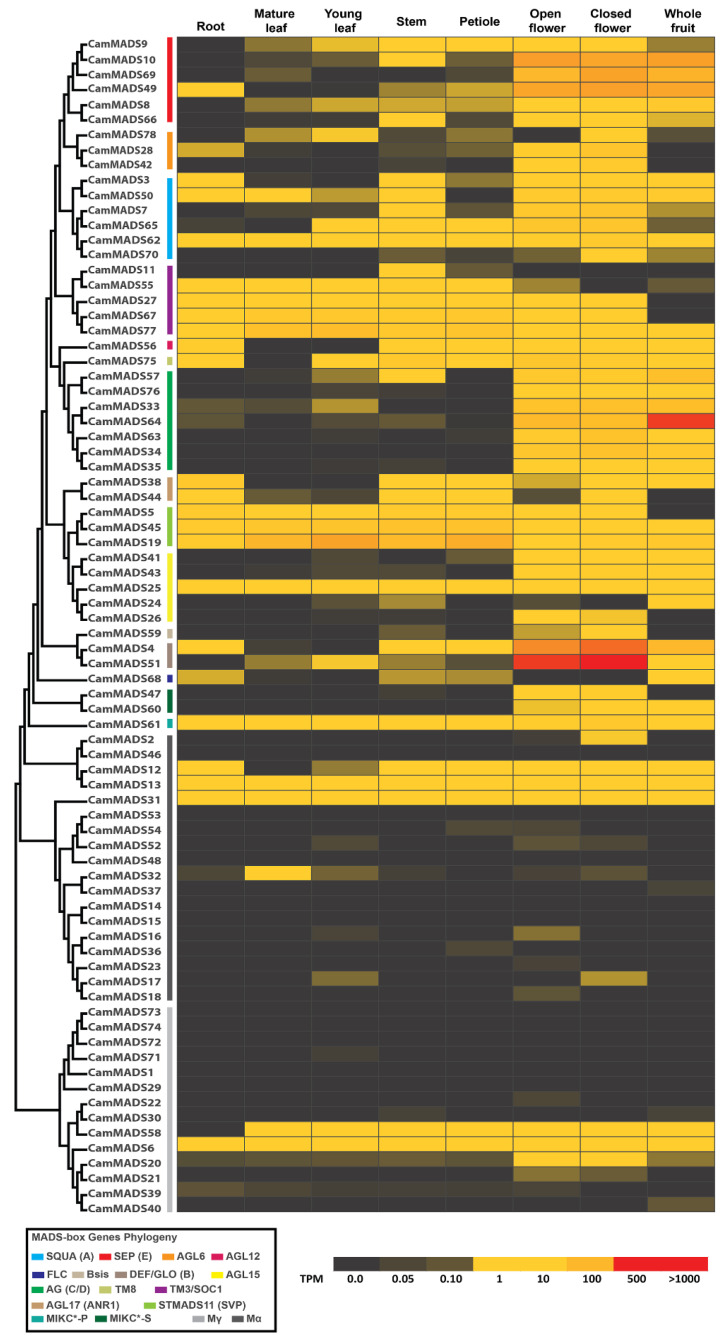
Heatmap of *CamMADS* genes expression level (TPM) is each of the following tissues: root, mature leaf, young leaf, stem, petiole, open flower, closed flower, and whole fruit. The phylogenetic tree is to the far left, and the box at the bottom left indicates the subgroups of *CamMADS* genes.

The expression profile heat map of the 78 *CamMADS* genes was generated using the transcript per million (TPM) data [2]. *CamMADS* genes expression was analyzed in the following tissues: mature leaf, young leaf, stem, petiole, root, open flower, closed flower, and whole fruit, as shown in Figure 6. Overall, the *CamMADS* genes were active in all plant tissues under study, indicating their versatile role in many key physiological activities. The type II (MIKC) *CamMADS* genes had higher expression in the floral organ and later fruit, some of which were strictly expressed in the floral organ, which is expected as they are key regulators of the florogenesis process. The expression pattern suggests that the ABCDE model of flower development is also conserved in *C. americana*.

The MIKC subgroup DEF/GLO (B) members *CamMADS51* and *CamMADS4* genes have the highest expression values in closed and open flower tissues. The SEP (E) subgroup has a high expression level in the open and closed flower as expected, in addition to the whole fruit. While, *CamMADS64,* a member of the AG (C/D) subgroup, has the highest relative expression value at whole fruit tissue. Most of the type I *CamMADS* genes have relatively very low to no expression (0 TPM) at most of the tested tissue samples, while few others have moderate expressivity in all tissues. Among the type I MADS-box genes in *C. americana*, *CamMADS6*, a member of Mγ sub-group, in addition to *CamMADS13* and *CamMADS31*, were expressed in all analyzed tissues. *CamMADS58* has a similar expression pattern except in roots. While, *CamMADS17* and *CamMADS20* genes were expressed in the flower bud tissues.

**Figure 7:**
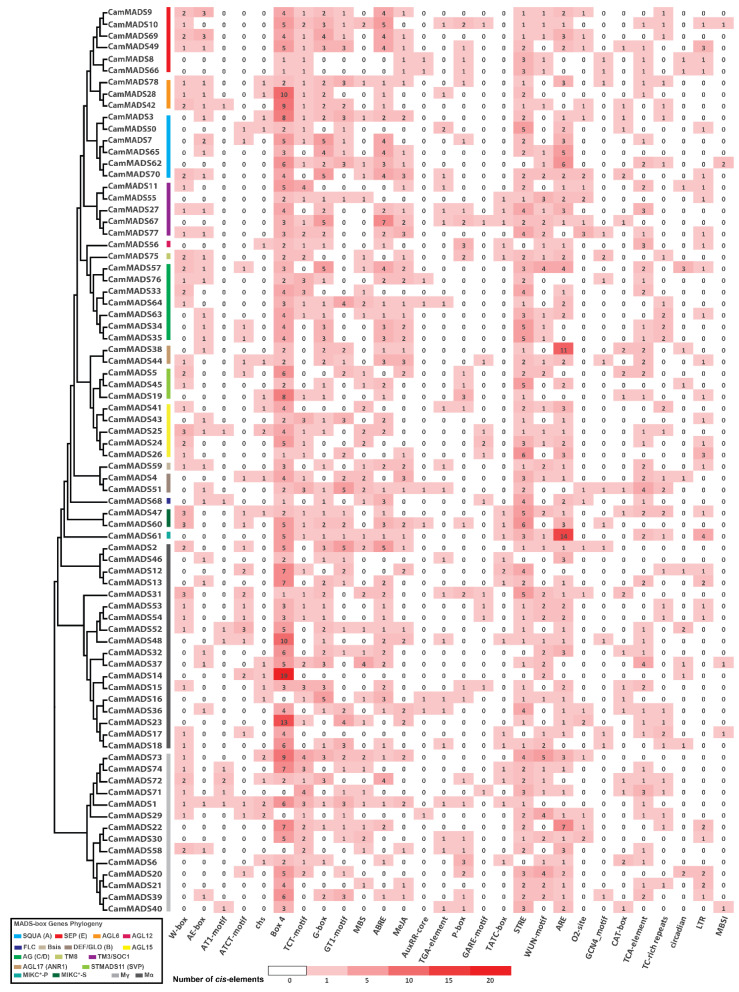
Analysis of *Cis*-acting elements of MADS-box gene family in *C. americana:* W-boxes, TC-reach repeats, AE-box, AT1-motif, chs, Box4, TCT-motif, G-box, GT1-motif, MBS, MYB binding site, ABRE, MeJA, AuxRR-core, TGA-element, P-box, GARE-motif, TATC-box, STRE, WUN-motif, ARE, O2-site, GCN4_motif, CAT-box, TCA-motif, Circadian, LTR, and MBSI.

To further assess the functions of *CamMADS* genes, the upstream 2 kb promoter region was analyzed for *cis*-acting regulatory elements, as shown in Figure 7. The following elements of key roles have been identified: W-boxes and TC-reach repeats are defense and stress-inducible promoters. AE-box, AT1-motif, chs, Box4, TCT-motif, G-box, and GT1-motif are involved in light responsiveness. MBS is the drought resistance-induced MYB binding site. ABRE is an abscisic acid response element. MeJA is the CGTCA-motif methyl jasmonate response element. AuxRR-core and TGA-element are regulatory auxin responsiveness elements. P-box, GARE-motif, and TATC-box are gibberellin response elements. STRE and WUN-motif are wound response elements. ARE is an anti-oxidant response element. O2-site is involved in zein metabolism regulation. GCN4_motif is involved in endosperm expression. CAT-box is related to meristem expression. TCA-motif is a salicylic acid response element. Circadian are cis-acting regulatory element involved in circadian control. LTR is involved in low-temperature responsiveness. MBSI is involved in flavonoid biosynthetic gene regulation.

All *CamMADS* genes have at least one regulatory element involved in light responsiveness. All have elements involved in wound response, except *CamMADS46*. Fifty-three genes have an abscisic acid response element. Forty-one genes have a methyl jasmonate response element. Twenty-six genes have either one or both of auxin responsiveness elements. Thirty-four genes have gibberellin response elements. Thirteen genes have an element involved in circadian control. Twenty-nine genes have elements involved in low-temperature responsiveness. A number of genes have different elements involved in metabolism and cell differentiation.

The upstream promoter regions were scanned for elements of GAGA (C-box), mainly GAGAGA hexamers and TGACGT-containing elements. Considering the other possible variation in the GA rich regions [35,36], all promoters had at least one of the aforementioned elements. In addition, all promoters have TATA-box and CAAT-box elements.

## 3. Discussion

MADS-box genes have been identified in several species, both the numbers and the types of MADS-box genes differed greatly among these species. Some species had very few type I (M-type) genes or lacked them totally, as in: *Saccharum officinarum* (grass), *Marchantia polymorpha* (Marchantiophyta), *Klebsormidium flaccidum, Dunaliella salina,* and *Chlorella variabilis* (Algaea). While, the Angiosperms species *Amaranthus hypochondriacus* and *Jatropha curcas* have ten genes. Several algae species had very few or lacked the type II (MIKC) genes, as in: *Bathycoccus prasinos, Chlamydomonas reinhardtii,* and *Volvox carteri. Marchantia polymorpha* (Marchantiophyta) has two type II (MIKC) genes, and *Picea abies* (Pinophyta) has three. While, the Angiosperms specie *Daucus carota* has five genes. Angiosperms also have the largest number of type I genes (*Camelina sativa*: 271 genes) and the largest number of type II genes (*Glycine max*, Soybean: 209 genes) [29,30].

The number of type I MADS-box genes in *C. americana* (32) was similar to *S. indicum* (31), but lower than *Ocimum tenuiflorum* (42), all members of Lamiaceae family. While, the number of type II genes in *C. americana* (46) was higher than that in *O. tenuiflorum* (43) but lower than *S. indicum* (62). The genome size of *C. americana* was 506.1 Mb [4], compared to the genome size of *S. indicum* 337 Mb [34] and 612 Mb estimated genome size for *O. tenuiflorum* [37]. When compared to the large soybean genome (1115 M) [34,38], which also has 269 MADS box genes, and *Camelina sativa* estimated the genome size of 785 Mb [39], which has 384 MADS box genes. The reduced number of genes in some Lamiaceae members might be justified by the smaller genome size and/or more active genome size reduction after duplication events, since the whole genome duplication event is a main contributor for the genes’ number increment and diversification of species [40,41,42,43]. The clustering of genes is observed in other transcription factor families, such as Hox genes [44]. This clusters might have risen through tandem gene duplication events [18,45]. The high exon number in type II (MIKC) genes (5–15) compared to type I (1–9) is consistent with studies in other species, such as sesame, *Arabidopsis*, rice, and soybeans [32,34,38]. This also matches the more complex and versatile functions found in type II (MIKC) compared to type I (M-type) [7,12,18,23]. 

The Mβ-type of type I MADS-box genes was absent in *C. americana*; also, it was absent in *S. indicum* and *U. gibba* [34]. The absence of Mβ-type genes in these species, which are all members of the Lamiales order, is an indication of a close relationship between the Lamiacaea family (*C. americana* and *S. indicum*) and Lentibulariaceae family (*U. gibba*) within the Lamiales order. The function of most Mβ-type genes in Arabidopsis is not fully understood, but some play important roles in the differentiation of female gametophyte [32,46]. Either there is a different mechanism in *C. americana* due to the lack of Mβ-type genes, or there was a redundancy in their function and other CamMADS protein can still fill their role in the protein network. Mβ genes were reported to be absent in rice and other monocots as well [32], and the subgroup might have evolved as a lineage-specific clade.

*CamMADS75* is an ortholog of *TM8* gene present in *S. lycopersicum*, *S. indicum*, and *A. trichopoda,* but absent in *A. thaliana*. TM8-like genes were identified in gymnosperms and angiosperms. The pattern of genes expression in several different tissues and the lack of a clear associated phenotype related to TM8 deletion or overexpression render it difficult to pinpoint an exact function, and it could indicate that TM8-like genes are a clade of fast evolving genes [31,47]. Its promoter region has elements involved in stress and drought response, jasmonate and gibberellin response elements, and the GCN4_motif, which is involved in endosperm expression. Further molecular and systematic analysis of *C. americana CamMADS75* TM8 ortholog could provide useful information on the function of this elusive gene.

In general, in each studied tissue, there was at least one *CamMADS* active gene being expressed. This hints to the importance and diversity in functions of this gene family in the *C. americana* plant. Type II *CamMADS* genes have an overall higher expressivity across all tissues compared to type I CamMADS. This is expected and can be justified, as the MIKC type genes are more complex and diverse than the M-type genes [7,12,18,23]. *CamMADS51,* an ortholog of *Arabidopsis PISTILLATA* (*PI*) gene, has the highest expression level in closed flower sample, along with *CamMADS4,* an ortholog of the *Arabidopsis APETALA3* (*AP3*) gene. This is reasonable for the key roles that *PI* and *AP3* plays during the florogenesis [19]. *CamMADS64* an ortholog of *Arabidopsis AG* gene was highly expressed in whole fruit sample [48,49,50]. *CamMADS68*, an ortholog of *Arabidopsis FLC*, was suppressed during flower development, since it is a suppressor of flowering, implying that it has a conserved function in *C. americana* [14,21,23]. *CamMADS47* and *CamMADS60*, members of the MIKC*-S subgroup, were expressed in flower tissues, hinting to a possible conserved function during male gametophyte development [15,18,22].

Some of the MIKC group genes were expressed in root, stem, and leaves tissues in addition to their key role in florogenesis. This is consistent with the patterns of MADS-box gene expression in *A. thaliana* where several genes are involved in biological processes other than florogenesis. *A. thaliana FLM* and *FLC* are involved in vernalization. *FLC*, SVP, and *SOC1* are involved in drought response; the presence of *cis*-acting regulatory elements in the promoter regions involved in drought response in ortholog *CamMADS* implies a possible conservation of functions. *ANR1* and *AGL21* are involved in lateral root formation; both respective ortholog *CamMADS38* and *CamMADS44* are expressed in the *C. americana* root. *SOC1*, *AGL21*, and *FLC* are involved in abscisic acid (ABA) and gibberellin (GA) metabolism [8]; their orthologs in *C. americana* have the *cis*-acting regulatory elements involved in ABA and gibberellin GA metabolism. These functions might be conserved in *C. americana* as well, for the orthologs expression profile can justify the presence of these subgroups’ members in the plants’ respective tissues.

All promoters had at least one of the GAGA (C-box) elements, which is required for the normal expression of a wide range of different genes; it can facilitate activation by a remote enhancer. Cytokinin response elements was shown to interact with the C-box in *A. thaliana* [51,52]; a similar mechanism could be at play here in *C. americana*.

In addition to the upstream promoter region, the first intron of each *CamMADS* genes—when available—was scanned for cis-regulatory elements, all introns contained TATA-box and/or CAAT-box elements, in addition to few other elements found in the upstream promoter region. This might point to a possible role of the intronic region in gene regulation in *CamMADS* genes [53].

In *A. thaliana*, most type I MADS-box genes are expressed weakly, and their function is not as clear as type II MADS-box genes. The expression of *CamMADS17* and *CamMADS20* genes in the flower bud tissues suggested that they might have a role in flower development. This is in line with what some studies suggest that type I genes are involved in *A. thaliana* reproduction and development [32,46]. It is worth noting that some genes appear to have no expression in any *C. americana* tissue. This might be due to the fact that some of the MADS-box genes are activated in response to certain environmental cues and abiotic stress responses, such as: temperature, salinity, drought, and wound response [8,9]. Another possibility is that these gens might be pseudogenes being transcribed to RNA at a very low level, with no function, or might be redundant genes going through neofunctionalization process. The presence of two or more orthologs of *A. thaliana MADS-box* genes either reflect a functional redundancy, or some of these genes might have acquired new functions, or they might differ in response to different environmental cues to fine tune gene expression level in *C. americana*. The *C. americana* genome analyses have revealed three putative whole-genome duplication events [2]. Gene duplication events were also recently reported in mints [43]. Whole genome duplication events might have contributed to MADS-box gene family expansion.

## 4. Conclusions

Based on the latest *C. americana* genome sequence and RNA-Seq data, 78 *CamMADS* genes were identified using bioinformatics tools and were classified as M-type (Mα and Mγ) and MIKC-type (MIKC* and MIKC^C^) according to their evolutionary relationships and protein structure characteristics. The Mβ-type of type I MADS-box genes was absent in *C. americana*, as it was absent in *S. indicum* and *U. gibba*. The absence of Mβ-type genes in these species, which are all members of the Lamiales order, might hint to a close relationship between Lamiacaea family and Lentibulariaceae family within the Lamiales order. Gene structure analysis revealed that type II genes contained a greater number of exons than did type I genes. The expression pattern of *CamMADS* genes in eight tissues, and the *cis*-regulatory element analysis of their promoter regions suggest an overall conservation of some of the abiotic stress responses and the ABCDE model of flower development functions to some extent in *C. americana.* The absence of certain elements and the change in expression patterns could point to some MADS-box genes being diversified in functions, or simply to a redundancy in function. This study will help guide future molecular protein–protein interaction analysis studies to confirm the interactions and functions of each of the *CamMADS* genes presented.

## 5. Materials and Methods

### 5.1. Identification and Sequence Analysis of MADS-Box Genes

The *C. americana* (beautyberry) genome and proteome were downloaded from NCBI (PRJNA529675). The hidden Markov model (HMM) profiles of the SFR (type I) domain (PF00319) and Myocyte Enhancer Factor-2 (MEF2) type II domain (PF09047) were retrieved from Pfam [54]. MADS-box genes were identified in the *C. americana* proteome using the hidden Markov model (HMM) profile corresponding to the Pfam MADS-box family PF00319 and PF09047 domains, using HMMER v. 3.0 [25], and redundant sequences were removed manually. A total of 78 MADS-box proteins were obtained as candidate MADS-box genes. The amino acid sequences were then searched, based on the conserved domains, using ScanProsite and the simple modular architecture research tool (SMART) to confirm that all genes contained the MADS-box domain [26,27]. The annotations of the type II (MIKC) genes that were missing the K-domain were corrected by the FGENESH suite [28,29], using the CamMADS genomic DNA in reference to the *S. indicum* genes. The online tool ProtParam [55] was employed to analyze theoretical molecular weights and isoelectric points (PI).

### 5.2. Assigning the Location of MADS-Box Genes to the C. americana Genome

The physical positions of MADS-box genes were mapped to the 17 chromosomes of *C. americana* using the coding DNA sequence files. The TBtool suite [56] was used to visualize the genes on chromosomes. The OrthoFinder algorithm [57] was used to identify possible duplicated genes.

### 5.3. Alignment and Phylogenetic Analysis of MADS-Box Genes

Type I and type II MADS-box proteins of *A. thaliana, S. lycopersicum, S. indicum*, and *A. trichopoda* were downloaded from PlantTFDB 5.0 database [30], then *C. americana* type I and type II MADS-box full length proteins were aligned to them using UGENE MUSCLE [58] with the following settings (Gap Open: −2.9, Gap Extended: 0.0, Hydrophobicity Multiplier: 1.2, Cluster Method: UPGMA). Two unrooted maximum likelihood (ML) trees of *C. americana, A. thaliana, S. lycopersicum*, and *S. indicum* type I and type II MADS-box proteins were constructed using the MEGA-X software [59,60] with the following settings (Bootstrap: 1000, Model: Jones-Taylor-Thornton, Uniform rates, Gaps: Use all sites). Another circular and linear phylogenetic tree of all *C. americana* MADS-box proteins was constructed using the same method.

### 5.4. Gene Structure and Conserved Motif Analysis

Gene structures were constructed using a GFF3 file downloaded from the GIGA database [61], BioProject (PRJNA529675). The structures were displayed using Gene Structure Display Server (GSDS 2.0) [62]. The MEME server [63] was used to predict conserved motifs with the following parameters: number of repetitions = any, maximum number of motifs = 20, optimum motif width set to ≥6 and ≤200, based on our knowledge of MADS-box protein domains.

### 5.5. Expression Profiling of MADS-Box Genes and Cis-Acting Regulatory Element Analysis

The RNA-Seq data were obtained from GIGA database [61] SRA study (SRP192973). These RNA-Seq data contained the transcriptomes of young leaf, mature leaf, stem, petiole, root, close flower, open flower, and whole fruit. Transcript abundance was calculated by normalized transcripts per million (TPM) values, and data were represented as a heatmap, using MS Excel sheets [64]. The *cis*-acting regulatory element analysis was performed on 2kb upstream promoter sequences of *CamMADS* genes, using PlantCARE server [65], and the number of elements was presented as a heatmap.

## Figures and Tables

**Table 1:** Detailed information for the MADS-box gene family in *C. americana*.

Gene Name	Gene ID	Chr	Exons	Length (aa)	PI	MW (Da)	Group	Ortholog	E-Value	Subgroup
*CamMADS1*	Calam.01G080200.1	01	1	119	8.58	14,045.36	M-type	*-*	-	γ
*CamMADS2*	Calam.01G122600.1	01	9	421	4.96	48,179.99	M-type	*-*	-	α
*CamMADS3*	Calam.01G172300.3 *	01	6	203	10.1	23,879.46	MIKC^c^	*-*	-	SQUA (A)
*CamMADS4*	Calam.01G249900.1	01	7	234	9.5	27,268.13	MIKC^c^	*AP3*	10^−90^	DEF/GLO (B)
*CamMADS5*	Calam.01G262900.1	01	8	227	5.92	25,470.73	MIKC^c^	*SVP*	10^−109^	STMADS11 (SVP)
*CamMADS6*	Calam.02G117500.1	02	1	389	7.99	44,905.13	M-type	*-*	-	γ
*CamMADS7*	Calam.03G039300.1	03	8	253	8.45	29,036.21	MIKC^c^	*CAL*	10^−107^	SQUA (A)
*CamMADS8*	Calam.03G039400.1	03	8	244	9.05	28,027.89	MIKC^c^	*SEP4*	10^−89^	SEP (E)
*CamMADS9*	Calam.03G072400.1 *	03	8	242	8.99	27,364.05	MIKC^c^	*SEP3*	10^−125^	FLC
*CamMADS10*	Calam.03G072500.1	03	8	242	8.99	27,574.33	MIKC^c^	*SEP3*	10^−127^	SEP (E)
*CamMADS11*	Calam.03G115700.1 *	03	5	161	9.3	18,451.68	MIKC^c^	*AGL14*	10^−46^	TM3/SOC1
*CamMADS12*	Calam.03G148300.1	03	1	212	6.94	23,923	M-type	*-*	-	α
*CamMADS13*	Calam.03G148400.1	03	1	183	4.96	20,172.29	M-type	*-*	-	α
*CamMADS14*	Calam.04G062300.1	04	1	314	9.53	34,355.29	M-type	*-*	-	α
*CamMADS15*	Calam.04G062400.1	04	1	127	10.03	14,061.52	M-type	*-*	-	α
*CamMADS16*	Calam.04G062600.1 *	04	1	216	9.11	24,304.77	M-type	*-*	-	α
*CamMADS17*	Calam.04G062900.1	04	1	99	8.96	10,888.37	M-type	*-*	-	α
*CamMADS18*	Calam.04G063300.1	04	1	346	5.17	37,570.27	M-type	*-*	-	α
*CamMADS19*	Calam.04G078200.1	04	9	268	9.32	30,383.48	MIKC^c^	*AGL24*	10^−48^	STMADS11 (SVP)
*CamMADS20*	Calam.04G086600.1	04	1	338	8.83	38,982.02	M-type	*-*	-	γ
*CamMADS21*	Calam.04G092200.1	04	1	339	8.99	39,032.72	M-type	*-*	-	γ
*CamMADS22*	Calam.04G128000.1	04	1	241	9.27	26,959	M-type	*-*	-	γ
*CamMADS23*	Calam.04G142100.1	04	1	295	9.57	32,333.33	M-type	*-*	-	α
*CamMADS24*	Calam.04G164100.1	04	7	270	5.19	30,133.31	MIKC^c^	*AGL15*	10^−39^	AGL15
*CamMADS25*	Calam.04G164200.1	04	8	232	5.01	26,355.97	MIKC^c^	*AGL15*	10^−29^	AGL15
*CamMADS26*	Calam.04G164300.1	04	8	332	5.37	37,578.7	MIKC^c^	*AGL15*	10^−45^	AGL15
*CamMADS27*	Calam.04G209300.1 *	04	5	169	9.39	19,122.03	MIKC^c^	*SOC1*	10^−59^	TM3/SOC1
*CamMADS28*	Calam.04G209500.1	04	8	251	8.91	28,497.25	MIKC^c^	*AGL6*	10^−103^	AGL6
*CamMADS29*	Calam.04G216300.1	04	1	262	7.04	30,161.8	M-type	*-*	-	γ
*CamMADS30*	Calam.04G237100.1	04	1	261	5.74	28,960.63	M-type	*-*	-	γ
*CamMADS31*	Calam.05G032500.1	05	1	215	8.88	24,307.89	M-type	*-*	-	α
*CamMADS32*	Calam.05G032800.1	05	1	248	5.35	28,277.95	M-type	*-*	-	α
*CamMADS33*	Calam.05G039300.2	05	7	277	9.26	31,920.44	MIKC^c^	*AG*	10^−117^	AG (C/D)
*CamMADS34*	Calam.05G230800.1	05	7	238	9.69	27,566.42	MIKC^c^	*SHP1*	10^−111^	AG (C/D)
*CamMADS35*	Calam.05G230900.2 *	05	9	282	9.41	33,043.92	MIKC^c^	*SHP1*	10^−89^	AG (C/D)
*CamMADS36*	Calam.06G036400.1	06	1	206	7.05	23,534.14	M-type	*-*	-	α
*CamMADS37*	Calam.06G036500.1	06	1	249	5.18	27,366.98	M-type	*-*	-	α
*CamMADS38*	Calam.06G039300.2 *	06	9	247	9.44	28,010.07	MIKC^c^	*ANR*	10^−80^	AGL17 (ANR1)
*CamMADS39*	Calam.06G055800.1	06	1	296	7.78	34,434.83	M-type	*-*	-	γ
*CamMADS40*	Calam.06G056500.1	06	1	294	7.72	33,540.55	M-type	*-*	-	γ
*CamMADS41*	Calam.06G225200.2	06	8	272	9.01	30,558.2	MIKC^c^	*AGL15*	10^−58^	AGL15
*CamMADS42*	Calam.06G255900.1	06	8	249	8.6	28,570.49	MIKC^c^	*AGL6*	10^−101^	AGL6
*CamMADS43*	Calam.07G213100.1	07	8	247	8.25	27,924.98	MIKC^c^	*AGL18*	10^−54^	AGL15
*CamMADS44*	Calam.08G005200.1	08	7	235	9.13	27,057.88	MIKC^c^	*AGL21*	10^−106^	AGL17 (ANR1)
*CamMADS45*	Calam.08G031600.1	08	8	228	6.56	25,831.27	MIKC^c^	*SVP*	10^−111^	STMADS11 (SVP)
*CamMADS46*	Calam.08G041400.1 *	08	8	388	5.08	44,294.51	M-type	*-*	-	α
*CamMADS47*	Calam.08G041600.1	08	11	329	5.57	36,829.16	MIKC*	*AGL104*	10^−60^	S
*CamMADS48*	Calam.08G166600.1	08	3	223	9.53	25,926.95	M-type	*-*	-	α
*CamMADS49*	Calam.09G141700.1	09	8	246	8.19	28,172.93	MIKC^c^	*SEP2*	10^−120^	SEP (E)
*CamMADS50*	Calam.09G141800.1	09	8	248	9.31	28,813.63	MIKC^c^	*AP1*	10^−64^	SQUA (A)
*CamMADS51*	Calam.10G107400.1	10	7	213	7.82	25,002.75	MIKC^c^	*PI*	10^−82^	DEF/GLO (B)
*CamMADS52*	Calam.10G116200.1	10	3	249	4.97	26,032.55	M-type	*-*	-	α
*CamMADS53*	Calam.10G116300.1	10	1	406	5.46	43,692.96	M-type	*-*	-	α
*CamMADS54*	Calam.10G116700.1	10	1	360	5.41	39,162.3	M-type	*-*	-	α
*CamMADS55*	Calam.10G171700.1	10	7	209	9.08	23,889.61	MIKC^c^	*AGL19*	10^−68^	TM3/SOC1
*CamMADS56*	Calam.11G009500.1	11	7	213	8.76	24,464.63	MIKC^c^	*AGL12*	10^−65^	AGL12
*CamMADS57*	Calam.11G010000.1	11	7	221	9.55	25,595.38	MIKC^c^	*STK*	10^−100^	AG (C/D)
*CamMADS58*	Calam.11G011000.1 *	11	1	182	9.38	20,612.54	M-type	*-*	-	γ
*CamMADS59*	Calam.11G041000.1	11	6	251	6.84	29,406.47	MIKC^c^	*ABS*	10^−51^	Bsis
*CamMADS60*	Calam.11G131900.1	11	11	326	5.5	37,186.12	MIKC*	*AGL66*	10^−54^	S
*CamMADS61*	Calam.12G155600.11	12	10	370	5.76	46,664.87	MIKC*	*AGL65*	10^−80^	P
*CamMADS62*	Calam.12G166700.1	12	8	236	9.11	27,139.8	MIKC^c^	*FUL*	10^−82^	SQUA (A)
*CamMADS63*	Calam.13G078400.1	13	7	244	9.17	28,250.83	MIKC^c^	*SHP1*	10^−105^	AG (C/D)
*CamMADS64*	Calam.13G161700.1 *	13	6	229	9.13	26,437.96	MIKC^c^	*AG*	10^−107^	AG (C/D)
*CamMADS65*	Calam.14G029500.1	14	8	245	7.65	28,377.37	MIKC^c^	*AP1*	10^−117^	SQUA (A)
*CamMADS66*	Calam.14G029600.1	14	8	245	9.18	27,993.7	MIKC^c^	*SEP4*	10^−82^	SEP (E)
*CamMADS67*	Calam.14G070200.1	14	7	210	9.32	24,190.64	MIKC^c^	*FYF*	10^−80^	TM3/SOC1
*CamMADS68*	Calam.14G138300.1 *	14	7	198	7.63	22,305.52	MIKC^c^	*FLC*	10^−34^	FLC
*CamMADS69*	Calam.14G138400.2	14	8	241	8.81	27,314.03	MIKC^c^	*SEP3*	10^−126^	SEP (E)
*CamMADS70*	Calam.15G063200.4	15	9	300	7.65	31,373.45	MIKC^c^	*FUL*	10^−94^	SQUA (A)
*CamMADS71*	Calam.15G133600.1	15	1	282	8.46	31,931.5	M-type	*-*	-	γ
*CamMADS72*	Calam.15G133700.1	15	1	260	6.37	29,363.63	M-type	*-*	-	γ
*CamMADS73*	Calam.15G135000.1	15	1	217	9.38	24,754.44	M-type	*-*	-	γ
*CamMADS74*	Calam.15G135100.1	15	1	218	9.52	24,769.46	M-type	*-*	-	γ
*CamMADS75*	Calam.16G114400.2	16	7	195	9.56	22,684.08	MIKC^c^	*TM8*	10^−85^	TM8
*CamMADS76*	Calam.17G013600.1	17	7	223	9.57	25,780.54	MIKC^c^	*STK*	10^−89^	AG (C/D)
*CamMADS77*	Calam.17G075300.1 *	17	7	211	9.28	24,291.01	MIKC^c^	*AGL71*	10^−59^	TM3/SOC1
*CamMADS78*	Calam.17G076100.1	17	7	194	9.64	22,181.55	MIKC^c^	*AGL13*	10^−48^	AGL6

* Gene annotation has been corrected. Chr = chromosome. *TM8* gene is present in *S. lycopersicum* but not in *A. thaliana*.

## Data Availability

Data is contained within the article or Supplementary Material.

## Supplementary Materials

The following are available online at www.mdpi.com/article/10.3390/plants10091805/s1, Figure S1: Phylogenetic maximum likelihood tree of Type I MADS-box proteins in *C. americana*, *A. thaliana*, *S. lycopersicum*, *S. indicum* and *A. trichopoda*. Figure S2. Phylogenetic maximum likelihood tree of Type II MADS-box proteins in *C. americana*, *A. thaliana*, *S. lycopersicum*, *S. indicum* and *A. trichopoda*.
