# Supplementary material for: Genome-Wide Identification and Analysis of the MADS-Box Gene Family in American Beautyberry (Callicarpa americana)
Source: Plants (Basel). 2021 Aug 30;10(9):1805. doi: 10.3390/plants10091805 (PMC8466759; doi:10.3390/plants10091805)
Supplement: Supplementary file 1 [file plants-10-01805-s001.zip › plants-1325603-supplementary/Supplementary Data.pdf]

Supplemetery Data

Genome-wide identification and analysis of the MADS-box gene family in American beautyberry (*Callicarpa americana*)

Tareq Alhindi [1]*, Ayed M. Al-Abdallat [2]

[1] Department of Biological Sciences, School of Science, The University of Jordan, Amman 11942, Jordan; e-t.alhindi@ju.edu.jo

[2] Department of Horticulture and Crop Science, School of Agriculture, The University of Jordan, Amman 11942, Jordan; a.alabdallat@ju.edu.jo

* Correspondence: t.alhindi@ju.edu.jo

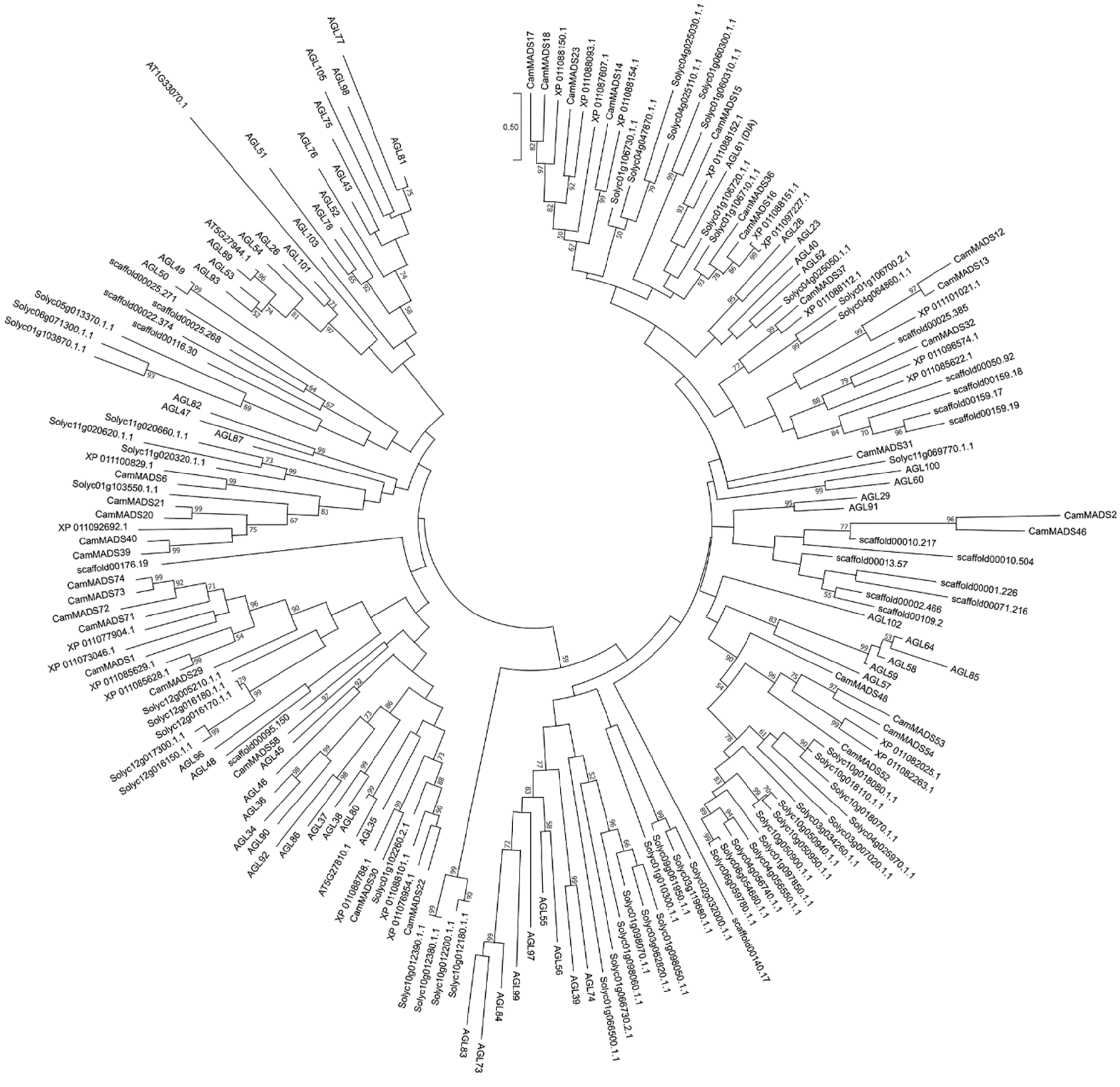

Supplementary Figure 1. Phylogenetic maximum likelihood tree of Type I MADS-box proteins in *C. americana*, *A. thaliana*, *S. lycopersicum*, *S. indicum* and *A. trichopoda*. The 1000 bootstrap values are presented, in addition to branch length with a scale.

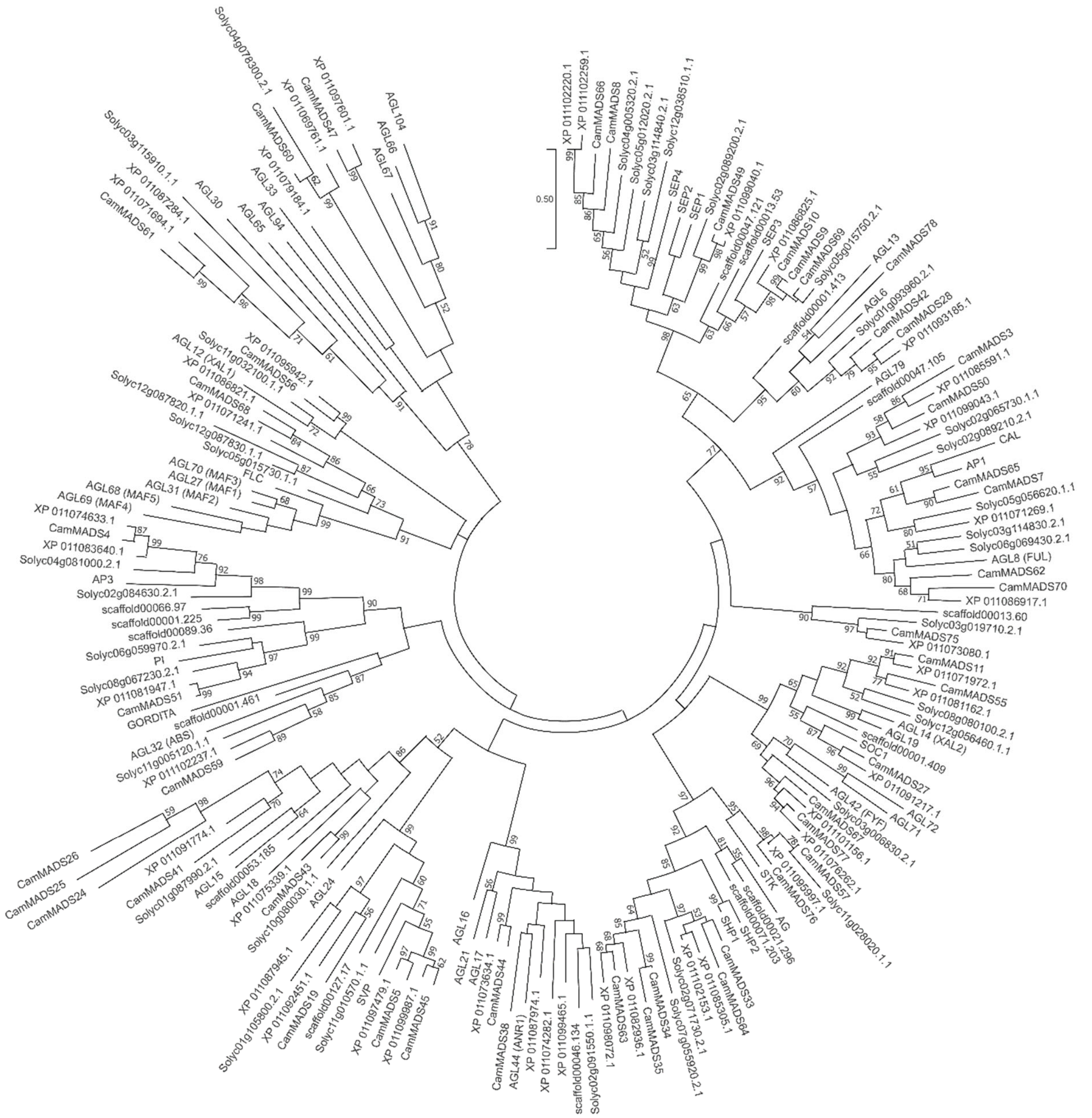

Supplementary Figure 2. Phylogenetic maximum likelihood tree of Type II MADS-box proteins in *C. americana*, *A. thaliana*, *S. lycopersicum*, *S. indicum* and *A. trichopoda*. The 1000 bootstrap values are presented, in addition to branch length with a scale.
